# LIECHTENSTEIN VERSUS LAPAROSCOPIC TRANSABDOMINAL PREPERITONEAL (TAPP) HERNIA REPAIR: A PROSPECTIVE COMPARATIVE STUDY FOCUSED ON POSTOPERATIVE OUTCOMES IN A GENERAL SURGERY UNIT

**DOI:** 10.1590/0102-672020210002e1642

**Published:** 2022-01-31

**Authors:** Carlos Augusto GOMES, Felipe Couto GOMES, Mauro PODDA, Ana Paula Fernandes BRAGA, Sarah Carvalho RIBEIRO, Larissa Fahel VAZ

**Affiliations:** 1 Hospital e Maternidade Therezinha de Jesus, Unidade de Cirurgia Digestiva - Juiz de Fora - Minas Gerais - Brasil; 2 University Hospital of Cagliari, Emergency Surgery - Cagliari - Sardinia - Itália; 3 Hospital de Clínicas da Unversidade Federal do Triângulo Mineiro, Unidade de Coloproctologia - Uberaba - Minas Gerais - Brasil; 4 Faculdade de Ciências Médicas e da Saúde (FCMS/JF) - SUPREMA, Medicina - Juiz de Fora - Minas Gerais - Brasil

**Keywords:** Hernia, Inguinal. General Surgery. Herniorrhaphy. Laparoscopy. Postoperative Complications, Hérnia Inguinal, Cirurgia Geral, Herniorrafia, Laparoscopia, Complicações Pós-Operatórias

## Abstract

Three surgical techniques for inguinal hernia repair are currently validated. Few studies have compared results among Lichtenstein and transabdominal preperitoneal (TAPP) laparoscopic approach obtained at an early step of the learning curve.

**AIM:**

This study aims to compare the early treatment results between the Liechtenstein technique and the laparoscopic TAPP approach to provide a basis for the surgeon’s decision-making.

**METHODS:**

Patients were divided into two groups: those who underwent laparoscopic TAPP approach (114 patients), and those who underwent open Lichtenstein repair (35 patients). Data were collected from the medical records during the evolution of the immediate postoperative period and by telephone contact after hospital discharge. For the analysis of the variables, the chi-square test of independence was implemented, with a level of significance set at a p-value of 0.05.

**RESULTS:**

There was a strong association between laparoscopy, less postoperative pain, and longer operative time. In addition, a preference for the technique in cases of recurrence, bilaterality, associated umbilical hernia, or obesity was noticed. In this study, the Lichtenstein technique was associated with a shorter time to return to work and was the treatment of choice for elderly patients.

**CONCLUSION:**

TAPP laparoscopic herniorrhaphy should be the first choice in cases of bilaterality, associated umbilical hernia, obesity, and recurrence to a previous anterior repair. The surgical risk is adequate for the procedure, even at early stages of the learning curve.

## RESUMO - RACIONAL

Três técnicas cirúrgicas para correção de hérnia inguinal estão atualmente validadas. Poucos estudos compararam os resultados entre Lichtenstein e a abordagem laparoscópica transabdominal pré-peritoneal obtidos em uma etapa inicial da curva de aprendizado.

**OBJETIVO:**

Comparar os resultados iniciais do tratamento entre a técnica de Liechtenstein e a abordagem pré-peritoneal transabdominal laparoscópica para fornecer uma base para a tomada de decisão do cirurgião.

**MÉTODO:**

Os pacientes foram divididos em grupo 1: aborgadem laparoscópica transabdominal pré-peritoneal (114 pacientes), e grupo 2: reparo aberto de Lichtenstein (35 pacientes). Os dados foram coletados em prontuários médicos durante a evolução do pós-operatório imediato e por contato telefônico após a alta hospitalar. Para a análise das variáveis, foi implementado o teste de independência Qui-Quadrado, com nível de significância estabelecido em p-valor = 0,05.

**RESULTADOS:**

Houve forte associação entre laparoscopia, menos dor pós-operatória e maior tempo operatório. Além disso, notou-se preferência pela técnica nos casos de recorrência, bilateralidade, hérnia umbilical associada ou obesidade. Neste estudo, a técnica de Lichtenstein foi associada a um menor tempo de retorno ao trabalho e foi o tratamento de escolha para pacientes idosos.

**CONCLUSÃO:**

A herniorrafia laparoscópica transabdominal pré-peritoneal deve ser a primeira escolha em casos de bilateralidade, hérnia umbilical associada, obesidade e recorrência para reparo anterior. O risco cirúrgico é adequado para o procedimento, mesmo nos estágios iniciais da curva de aprendizado.

## INTRODUCTION

Inguinal hernias result from points of less resistance in the aponeurotic muscle, which is contained in the myopectineal ring. Currently, operative treatment is recommended only in symptomatic cases, contrasting to the old aphorism, in which “diagnosed hernia is equal to operated hernia”^18^. In this context, surgical correction of inguinal hernias is the most commonly performed surgical procedure worldwide^4^.

Several approaches have been proposed over time for the treatment of inguinal hernias. However, only three surgical techniques are currently validated, including Shouldice technique, Lichtenstein technique, and laparoscopic techniques, such as transabdominal preperitoneal (TAPP) hernioplasty and totally extraperitoneal endoscopic hernioplasty^4^.

All these three techniques show specific advantages and disadvantages. The Liechtenstein technique, described in 1984, is tension free and is based on the insertion of a polypropylene mesh. Its advantages are easy learning, less operating time, a low recurrence rate (1%), and low cost^1^ ^,^ ^3^. Furthermore, it is the most widely used and validated surgical procedure for treating inguinal hernias^4^ ^,^ ^16^.

The advent of minimally invasive surgery and better knowledge of the anatomy and physiology of the posterior inguinal region allowed the development of the laparoscopic approach. It has been reported that laparoscopy enables faster recovery, a shorter time to return to work (TRTW), reduced pain, and a lower incidence of surgical site complications^4^ ^,^ ^19^.

Thus, it is crucial for teams who propose herniorrhaphy to select which patient is the most suitable for the procedure, especially mastering the unknown challenges encountered during its performance. Advanced age, comorbidities, major abdominal wall defect, bilaterality, recurrence, an extension of the hernial sac, previous pelvic surgery, and associated morbidities must be adequately considered and not underestimated. These aspects add responsibility due to the existence of an alternative that has been validated for decades. Finally, the profile of the initial patient candidate for the laparoscopic approach is questioned, in addition to considering the experience of the surgical team^6^.

Few prospective studies have approached the problem in order to show and discuss the difficulties and technical alternatives, what lessons have been learned, and what pitfalls should be avoided.

Therefore, this study aims to compare the early treatment results between the Liechtenstein technique and the laparoscopic TAPP approach to provide a basis for the surgeon’s decision-making.

## METHODS

This is an observational, prospective study conducted at Monte Sinai Hospital, Juiz de Fora, Minas Gerais, from 2014 to 2016. The study protocol was approved by the Research Ethics Committee of the Federal University of Juiz de Fora (CEP/UFJF, nº 21221714.0.0000.5133). The research implied a minimal risk to the participants, that is, there was no interference from the researcher in any aspect of the patient’s care. Furthermore, all patients expressed voluntary consent to participate and signed an informed consent form.

The study sample was nonprobabilistic and was selected based on the availability of the Digestive System Surgery Service of the referred institution. The surgeries were performed by three surgeons with at least 25 years of practice in minimally invasive surgery. All surgeries started with the laparoscopic approach via TAPP. The data were collected from the medical records of the immediate postoperative period (10 days) and telephone contact after hospital discharge (30 days). At the second contact with the patients, the researchers did not know the type of operation performed. The early results of operative treatment were then compared between the Lichtenstein technique and the laparoscopic technique (TAPP).

The adopted inclusion criteria were patients of any gender, aged above 18 years, with a clinical diagnosis of inguinal hernia and Nyhus II to IV classification. Patients were divided into two groups: those who underwent laparoscopy, and those who underwent open anterior Lichtenstein technique.

The studied variables were age, laterality, Nyhus classification, presence of an umbilical hernia, body mass index (BMI) value and stratification, operative time, intraoperative complications, postoperative complications at the surgical site, pain intensity, hospital permanence, and TRTW.

Intraoperative complications were defined as bowel perforation, parietal or intra-abdominal bleeding, thrombosis of the pampiniform plexus, and nerve damage. Complications of the surgical site were seroma, bruise, and infection. Surgical site infection was diagnosed clinically if hyperemia or purulent secretion was observed up to postoperative day 30. Diagnosis of intra-abdominal infection was suspected clinically and confirmed by an imaging method. The pain was assessed using Wong-Baker visual analog scale, in which the patients choose the one that best describes their level of pain. Hospital permanence was measured in hours from the moment the patient was admitted to hospital discharge. Operative time was measured in minutes from the first skin incision to the synthesis of the last skin suture.

Descriptive and exploratory statistics on the data were performed using absolute frequencies (n), relative frequencies (%), measures of central tendency, and measures of dispersion (standard deviation). For comparative analysis of the characteristics of the dichotomous qualitative variables, 2 × 2 contingency tables were generated containing the absolute and relative frequencies. The chi-square test of independence was performed without correction to assess the association between the variables. A 95% confidence interval (CI) was established, with p-value <0.05 being considered statistically significant.

The results involve statistically significant variables and clinically important variables for choosing the surgical technique (either laparoscopic or Liechtenstein), but without significant statistical effect in this study. The statistical software SPSS version 21.0^®^, 2015, was used for the statistical analysis and assembly of the database.

## RESULTS

A total of 149 patients were included, with a mean age of 54.6 ± 17 years and a median age of 55 years. Of these, 114 patients were operated by laparoscopy and 35 by Lichtenstein; 71.4% were above 55 years.

There was no relationship between laterality (i.e., right, left, and bilateral) and choice of the operative approach (p = 0.38). However, among bilateral hernias, 85.3% were operated by laparoscopy. Only 124 patients were classified according to Nyhus, with 50.8% being IIIa. Of these, 82.2% were operated by laparoscopy. However, there was no relationship between Nyhus classification and the chosen approach (p = 0.605). In 88.4% of the cases of associated umbilical hernia, the laparoscopic technique was preferred (p = 0.030).

The mean BMI was 25.7 ± 4.2 and median was 25.5, with 92.1% of the patients having a BMI up to the overweight range. There was no association between the surgical approach and the BMI (p = 0.846). However, of the 11 obese patients, 9 (81.8%) were operated by laparoscopy.

Among the 35 patients treated with the open Lichtenstein technique, 71.4% underwent surgery in <90 min. The laparoscopic technique was responsible for 83.1% of the operative time >90 min (p = 0.047).

Of the 85 patients with <24 h of hospital permanence, 81.2% were operated by laparoscopy. Among the patients operated on by the Lichtenstein technique, 54.3% were discharged after 24 h (p = 1.122).

A total of 112 participants were analyzed regarding the TRTW. Of note, 54.5% returned to work within 15 days. In the Lichtenstein group, 78.9% of patients returned to work within 15 days. In contrast, in the laparoscopy group, 50.5% returned to work after 15 days (p = 0.019).

Of the 149 study patients, no pampiniform plexus thrombosis or nerve injury in the inguinal region was identified. There was no relationship between the type of surgical approach and intraoperative bleeding (p = 0.43), the occurrence of postoperative seroma (p = 0.670), and bruises (p = 0.840). There was no report of surgical site infection in the sample.

There was an association between seroma and obesity (p = 0.005). Of the patients who had seroma, 71% were in the BMI range above overweight. In addition, bruises were more prevalent in right or bilateral laterality (p = 0.043).

The prevalence ratios of the variables analyzed between the laparoscopy and the Lichtenstein groups are listed in Table 1.

**Table 1 -:** Prevalence ratio between Lichtenstein and laparoscopy.

	Lichtenstein		Laparoscopy		Total	p	RCP	95% CI
	n^1^	%	n^1^	%				
Laterality								
Left	41	73.2	15	26.8	56	0.383		
Right	44	26.8	15	25.4	59			
Bilateral	29	85.3	5	14.7	34			
BMI								
Up to 24.9	14	23.7	45	76.3	59	0.729	0.867	0.388-1.938
>25.0	17	21.3	63	78.8	80			
Weight (kg)								
Up to 75	19	23.8	61	76.3	80	0.752	0.881	0.402-1.931
>75	14	21.5	51	78.5	65			
Age* (year)								
Up to 55	10	13.3	65	86.7	75	**0.003**	3.316	1.458-7.543
>55	25	33.8	39	66.2	74			
Sex								
Female	8	30.8	18	69.2	23	0.335	0.633	0.248-1.613
Male	27	22.0	96	78.0	123			
Top* (min)								
Up to 90	25	27.8	65	72.2	90	**0.047**	2.408	1.010-5.070
> 90	10	16.9	49	83.1	59			
Drained seroma								
Yes	1	20.0	4	80.0	5	0.814	0.764	0.080-7.263
No	17	16.0	89	84.0	106			
Seroma								
Yes	1	14.3	6	85.7	7	0.670	1.213	0.138-10.700
No	18	16.8	89	83.2	107			
Bruise								
Yes	3	15.0	17	85.0	20	0.840	1.148	0.301-4.382
No	16	16.8	79	83.2	95			
PHP (h)								
Up to 24	16	18.8	69	81.2	85	1.122	1.821	0.848-3.908
>24	19	29.7	45	70.3	64			
Umbilical hernia*								
**Yes**	5	11.6	38	88.4	43	**0.030**	3.000	1.078-8.350
**No**	30	28.3	76	71.7	106			
RT time* (day)								
Up to 15	15	24.6	46	75.4	61	**0.019**	0.261	0.081-0.846
>15	4	7.8	47	92.2	51			
Parietal bleeding								
Yes	2	100	0	0	2	0.430		
No	112	76.2	35	23.8	147			

*Significant values. **Values close to significance. n^1^ denotes total per column. BMI: body mass index, CI: confidence interval, PHP, RCP, RT: room temperature.

A total of 114 patients were assessed for pain. Of these, 61.1% were in pain up to 7 (moderate). Of the patients operated by laparoscopy, 26.3% were in the pain range 0, making up 83.3% of the patients without pain. Only 26.3% of the Lichtenstein group rated pain at 0 (p = 0.025). The moderate-to-severe pain complaint was more concentrated in the BMI of overweight and obesity (p = 0.03) (Table 2).

**Table 2 -:** Prevalence ratio between the grades of obesity.

	Normal weight		Overweight		Obesity 1		Obesity 2		Obesity 3		Total	p	RCP	95% CI
	n^1^	%	n^1^	%	n^1^	%	n^1^	%	n^1^	%				
Seroma*														
Yes	2	28.6	4	57.1	0	0.0	1	14.3	0	0.0	7	0.005		
No	43	43.9	47	48.0	5	5.1	0	0	3	3.1	98			
No pain*	14	46.7	14	46.7	2	6.7	0	0	0	0	30	0.030		
Intense pain	9	45	8	40	0	0	0	0	3	15	20			

*Significant values. *Values close to significance. n^1^ denotes total per column. CI: confidence interval.

## DISCUSSION

The Lichtenstein technique is indicated under local or spinal anesthesia in patients at high risk for general anesthesia^7^. The elderly patient is generally more susceptible to the cardiopulmonary depressive effects of preanesthetic and common anesthetic agents^11^. Therefore, each patient must be evaluated individually, but there is a preference for the open technique for the elderly. This was portrayed in this study, in which the majority of patients in the Lichtenstein group aged above 55 years.

In primary unilateral inguinal hernia in men and women and bilateral cases, the laparoscopic approach (e.g., totally extraperitoneal endoscopic hernioplasty and TAPP) is the first choice since the surgeon has experience^3^. In this series, there was no relationship between laterality (i.e., right, left, and bilateral) and the choice of surgical approach (p = 0.38). However, we have identified a tendency toward laparoscopy among bilateral hernias, a relevant fact for clinical practice, as the literature indicates this technique for repairing bilateral hernias^2^ ^,^ ^10^.

There was also a strong association between bruises formation and bilaterality, which can be justified by the wider dissection, compared with unilateral hernia repair^15^.

The Nyhus classification had no statistical evidence when compared between the two groups. Data that interfere in the choice of the surgical approach regarding the definition of hernias either directly or indirectly were not found in the literature.

Our study did not evaluate recurrent hernias. However, it is worth mentioning that the Guideline of the International Hernia Society indicates laparoscopy in case of recurrence after previous open repair^3^ ^,^ ^12^. Conversely, as for laparoscopic repair recurrence, it indicates the Lichtenstein technique.

In this study, there is a preference for laparoscopy in associated umbilical hernia, a situation also found in the literature review. The trocar is introduced through the hernia ring, followed by repair a second time by suture^9^.

Few studies compare the results of the laparoscopic versus open approach in obese patients. When analyzed, more favorable results are seen in the laparoscopy group. The difference is probably related to the greater subcutaneous dissection in open herniorrhaphy^7^. We opted for laparoscopy in 81.8% of obese patients.

In addition, we observed a strong association between higher BMI and more significant postoperative pain and the formation of seroma, which can be justified by the widespread retroperitoneal dissection created in a laparoscopic repair. This surgical approach tends to be more laborious in obese patients and, therefore, exposing this patient population to these adverse outcomes^7^.

In a systematic review, Bittner et al.^4^ identified the complexity of the technique as a disadvantage of laparoscopic inguinal herniorrhaphy, implying a risk of complications when the surgeon has not yet overcome his learning curve^4^ ^,^ ^8^. Thus, studies show that posterior repair results in a longer operative time, as occurred in this study.

Both laparoscopy and Lichtenstein proved to be safe techniques. Only two patients in the study had intraoperative bleeding, promptly controlled employing hemostatic clips. Both cases were in the laparoscopy group. However, there was no statistical evidence of greater risk in this group. The less experience of the surgeon may be associated with a higher risk of complications. However, in several studies, it has been demonstrated that in experienced hands, TAPP and totally extraperitoneal endoscopic hernioplasty are safe and practical techniques for treating inguinal hernias^4^ ^,^ ^17^.

Meta-analysis of randomized controlled trials, which compare laparoscopic inguinal herniorrhaphy versus conventional open technique, concluded that postoperative pain is similar between groups. However, the analysis of the pain profile is favorable to laparoscopy when compared to the previous repair^5^. In our study, most patients assessed pain in the absence of pain, mild pain, and moderate pain, regardless of the type of surgery. It is also noted that the highest frequency of pain is in the laparoscopy group, which is in accordance with studies that show less pain in the immediate postoperative period in these patients. Such favorable results may facilitate patients’ mobilization and early discharge. However, there was no statistical significance in this study regarding the assessment of hospital permanence^13^.

Finally, in our series, Lichtenstein repair was associated with less TRTW, which goes against the scientific literature, where these results are justified by the larger surgical incision and later discharge compared to laparoscopy^14^. However, contrary to what has been done in other studies, the types of employment relationships have not been separated. In addition, the team’s insecurity regarding less experience with laparoscopy may have influenced the analysis of the variable. However, with this study and in accordance with the literature, it appears that the technique is safe and has an excellent early prognosis. Therefore, it is possible to guide a shorter return time to work activities.

## CONCLUSIONS

Since no single technique is widely indicated for all inguinal hernias, the surgeon must consider the anterior (Lichtenstein) or posterior (TAPP or totally extraperitoneal endoscopic hernioplasty) repair, individualizing each patient. This study shows that both laparoscopic and Lichtenstein hernia repair are safe techniques. With extra care, in TAPP, intraoperative complications can be avoided.

Therefore, laparoscopy is the choice in recurrence, bilateralism, associated umbilical hernia, or obesity. Therefore, Lichtenstein is a choice for the elderly.

Pain is mild to moderate in both techniques, but patients undergoing laparoscopy have less pain in the immediate postoperative period, associated with earlier mobility and possible anticipation of discharge.

This study also shows that it is possible to reach high standards, even during the surgeon’s learning phase, if strict adherence to the protocols is pursued. Based on these initial experiences, hernia repair via laparoscopic TAPP may be the first choice in primary hernia repair in patients without comorbidities, without previous pelvic surgery, bilaterality, associated umbilical hernia, obesity, and recurrence to a previous anterior repair, as recommended by the guidelines of the International Endohernia Society.
